# Effect of different inner pressures of air insoles and walking durations on plantar pressure time integral

**DOI:** 10.1038/s41598-024-70312-x

**Published:** 2024-08-20

**Authors:** Gilang Titah Ramadhan, Fahni Haris, Yih-Kuen Jan, Ben-Yi Liau, Wen-Thong Chang, Chien-Cheng Tai, Chi-Wen Lung

**Affiliations:** 1 Department of Computer Science and Information Engineering, Asia University, Taichung, 413305 Taiwan; 2 School of Nursing, Universitas Muhammadiyah Yogyakarta, Yogyakarta, 55183 Indonesia; 3 Rehabilitation Engineering Lab, Department of Kinesiology and Community Health, University of Illinois at Urbana-Champaign, Champaign, IL 61820 USA; 4 Department of Automatic Control Engineering, Feng Chia University, Taichung, 407102 Taiwan; 5 School of Public Health, Taipei Medical University, New Taipei City, 235603 Taiwan; 6 Department of Creative Product Design, Asia University, Taichung, 413305 Taiwan

**Keywords:** Pressure time integral, Air insole, Walking duration, Insole inner pressure, Diabetic foot ulcer, Peripheral vascular disease, Trauma, Type 2 diabetes

## Abstract

Air insoles have provided insights for reducing the risk of diabetic foot ulcers (DFU). The pressure time integral (PTI) is an effective assessment that considers the time effect in various physical activities. We investigated the interactions between three different insole inner pressures (80, 160, and 240 mmHg) and two walking durations (10 and 20 min). The big toe (T1), first metatarsal head (M1), and second metatarsal head (M2) were investigated in 13 healthy participants. One-way analysis of variance (ANOVA) showed that the effects of each insole inner pressure significantly differed (*P* < 0.05) with a 10 min walking duration. The PTI values resulting from 80 mmHg in M2 (38.4 ± 3.8, *P* = 0.002) and 160 mmHg in M1 (44.3 ± 4.3, *P* = 0.027) were lower than those from 240 mmHg. Additionally, the paired t test showed that the effects of each walking duration were also considerably different at 160 mmHg. The PTI at 10 min was lower than that at 20 min in M1 (44.31 ± 4.31, *P* = 0.015) and M2 (47.14 ± 5.27, *P* = 0.047). Thus, we suggest that walking with a pressure of 160 mmHg for 10 min has a lower risk of DFU.

## Introduction

Diabetes mellitus (DM) is a chronic metabolic disorder characterized by elevated blood glucose levels resulting from either insufficient insulin production or inability of the body to effectively use insulin^1^. In 2022, the cost of treating diabetes in the US was estimated to be $412.9 billion annually. The cost is projected to increase to $494 billion by 2030, driven by the growing number of diabetes cases and associated healthcare expenditures^2^. DM is predicted to affect 783 million individuals by 2045^3^. Hence, policymakers must take urgent action to prepare health and social security systems to mitigate the impact of DM. One preventive intervention for DM is exercise, which may promote health and reduce the risk of chronic complications in patients with DM^4^. Walking is the most common exercise among individuals with DM^5,6^. Walking in individuals with diabetes mellitus may increase the risk of plantar skin breakdown due to repetitive high vertical and shear pressures on the plantar tissue, which can increase the risk of ulcers^7^. Previous studies have reported that wearing an inappropriate insole can increase the peak pressure and thus lead to the risk of diabetic foot ulcers (DFU)^8–11^.

Recent advances in the development of air insoles have demonstrated important improvements in mitigating discomfort and decreasing the risk of DFU^12^. Air insoles enclose air within a flexible bag in the shoe, increasing the shock absorption capacity and providing superior stability and comfort during ground contact^13^. This evidence points to the multifaceted benefits of air insoles, emphasizing their importance in promoting foot health and facilitating more effective exercise routines, particularly in people with DFU.

The hardness of the insoles is a crucial factor in foot health and injury prevention. Different hardness levels can significantly affect plantar pressure distribution, comfort, and muscle activity, thereby affecting the overall effectiveness of insoles in reducing foot injuries^14^. Suitable insole hardness plays a vital role in reducing the risk of DFU^10,15^. This is because variable hardness levels in insoles are instrumental in effectively redistributing pressure, as highlighted by Haris et al.^9^. Furthermore, the optimal insole hardness was greater than that obtained with softer insoles. The effectiveness of softer insoles in reducing peak plantar pressure (PPP) has been noted for their significance in preventing injuries, such as metatarsal problems and blisters^16^. In addition, longer walking durations have been associated with an increased complexity index of plantar soft tissues, suggesting potential alterations in their structure and function^17^. This heightened complexity may render tissues more susceptible to damage, thereby elevating the risk of developing DFUs.

Many studies have commonly used PPP to predict the risk of DFU based on the experience of the highest pressures during walking or weight-bearing activities^9,18–20^. Compared with PPP, the pressure time integral (PTI) is a more effective assessment tool for evaluating injuries or damage that considers time factors, such as tissue breakdown or ulceration^7^. Soames^21^ conducted the first study on PTI, integrating pressure over the entire stance phase, and concluded that combining information on pressure distribution and contact time provided a more comprehensive understanding of plantar mechanical loading^21^. The amount of pressure applied and duration of sustained pressure on the foot are crucial factors in assessing the load that causes prolonged pressure. This refers to the extended duration during which pressure is exerted on a particular area, potentially leading to increased strain or discomfort^22^.

Prolonged pressure, as quantified by PTI, can exacerbate these risks by causing damage to the skin and underlying tissues, potentially leading to ulcers^23^. Individuals with DM are at a higher risk of foot ulcers due to neuropathy and altered foot biomechanics, leading to abnormal plantar pressure distribution during walking^20^. Thus, studying PTI offers valuable insights into preventive strategies to reduce the risk of ulceration. This can be achieved by identifying and modifying the factors that contribute to excessive mechanical stress on the foot.

This study investigated the effects of different insole pressures and walking durations on PTI. The results of this study can be employed to optimize the air insole design for redistributing the plantar PTI in healthy patients. In addition, the results can also provide a foundation for understanding the effect of insole inner pressure and walking duration on PTI in individuals with DM and peripheral vascular disease. The use of healthy subjects in this study is justified, as it aims to establish baseline data and control conditions that are free from the confounding variables associated with DFUs. Research has shown that interventions aimed at preventing foot ulcers benefit from a robust understanding of plantar biomechanics and pressure distribution, which can be effectively studied in healthy populations before being applied to those at risk for DFUs^24^. Furthermore, studies have shown that biomechanical analyses do not always require non-insole baseline data, as interventions can be effectively evaluated under controlled, comparative conditions^25^.

We hypothesized that different interactions between the insole inner pressure and walking duration would influence the risk of DFU. Accordingly, various combinations of insole inner pressure and walking duration may generate distinct PTI values characterized by altered patterns of plantar pressure distribution over time and space. It has been recognized that such alterations have the potential to initiate biomechanical adaptations within the foot, which are conducive to reducing the negative effects caused by prolonged pressure exposure, an underlying cause of DFU. Therefore, a comprehensive examination of how various insole inner pressures and walking durations intricately interact to obtain PTI values could provide invaluable insights into the mechanisms underlying preventive strategies aimed at alleviating the risk of DFU occurrence. Therefore, this study aimed to determine which walking condition is suitable based on the effects of the insole inner pressure and walking duration on PTI to reduce the risk of DFU.

## Results

The effects of different insole inner pressures and walking durations, both individually and in combination, on PTI were analyzed using a (3 × 2) factorial multivariate analysis of variance (MANOVA). The results revealed no significant differences between individual and combination factors.

### Effect of insole inner pressures

**Figure 1:**
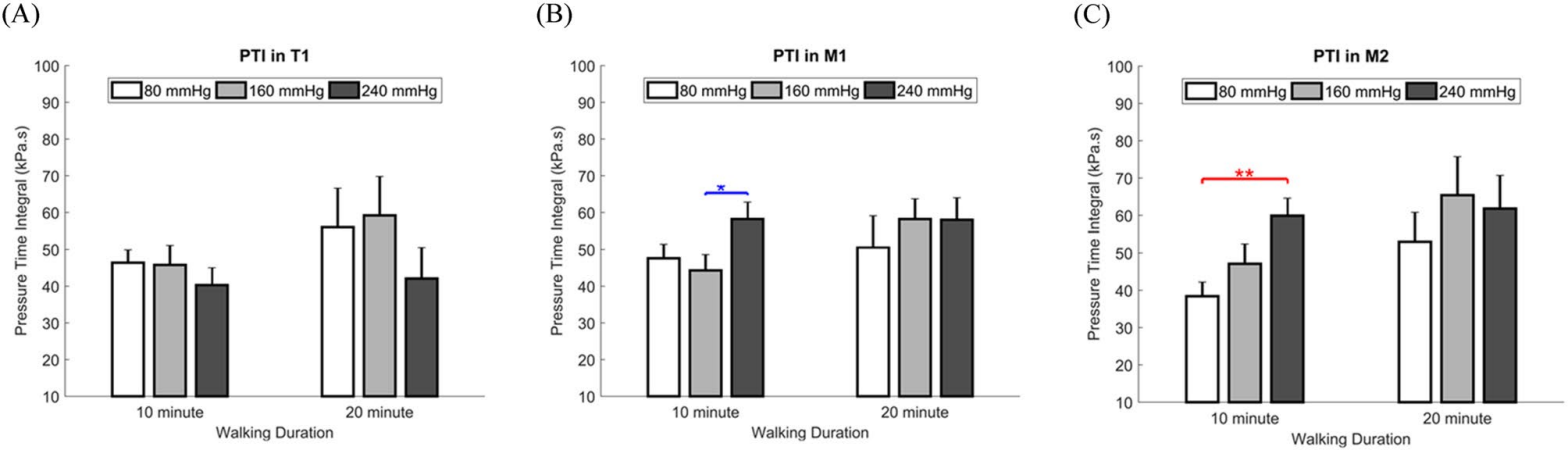
Comparisons of the effect of the insole inner pressures on the PTI at two walking durations. (A) Effect of the insole inner pressures on the PTI in T1; **(B)** Effect of the insole inner pressures on the PTI in M1; **(C)** Effect of the insole inner pressures on the PTI in M2. Data are shown as mean ± standard errors. PTI, pressure time integral; T1, first toe; M1, first metatarsal head; M2, second metatarsal head. *a significant difference (*P* < 0.05); **a significant difference (*P* < 0.01).

The results of the one-way analysis of variance (ANOVA) showed that the impacts of each insole inner pressure on the PTI were significantly different (Table 1 and Fig. 1). It demonstrated that, with a walking duration of 10 min, the PTI obtained with the insole inner pressure of 80 mmHg was considerably lower than that attained with 240 mmHg in M2 (38.4 ± 3.8 vs. 59.9 ± 4.7 kPa·s, *P* = 0.002). Meanwhile, the insole inner pressure of 160 mmHg obtained lower PTI than 240 mmHg in M1 (44.3 ± 4.3 vs. 58.2 ± 4.6 kPa·s, *P* = 0.027).

**Table 1:** Effect of insole inner pressure on the PTI.

Region	Walking duration	Insole inner pressure			One-way ANOVA	LSD Post hoc		
		80 mmHg (Mean ± SE)	160 mmHg (Mean ± SE)	240 mmHg (Mean ± SE)	***P*** value	80 mmHg versus 160 mmHg	80 mmHg versus 240 mmHg	160 mmHg versus 240 mmHg
T1 (kPa s)	10 min	46.4 ± 3.5	45.8 ± 5.3	40.3 ± 4.7	0.612	0.930	0.350	0.396
	20 min	56.0 ± 10.6	59.2 ± 10.6	42.1 ± 8.4	0.159	0.818	0.327	0.228
M1 (kPa s)	10 min	47.6 ± 3.8	44.3 ± 4.3	58.2 ± 4.6	0.040*	0.593	0.085	0.027*
	20 min	50.6 ± 8.5	58.2 ± 5.5	58.0 ± 6.0	0.589	0.434	0.447	0.982
M2 (kPa s)	10 min	38.4 ± 3.8	47.1 ± 5.3	59.9 ± 4.7	0.639	0.194	0.002**	0.060
	20 min	53.0 ± 7.8	65.4 ± 10.3	61.8 ± 8.9	0.712	0.337	0.495	0.778

Data are presented as the mean ± standard error. T1, first toe; M1, first metatarsal head; M2, second metatarsal head; *significant difference (***P*** < 0.05); **significant difference (*P* < 0.01).

### Effect of walking duration

**Figure 2:**
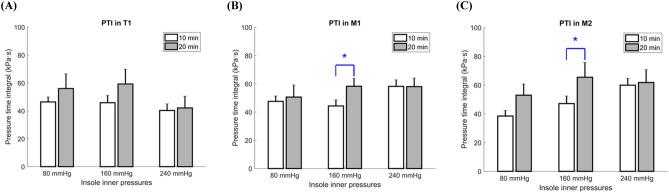
Comparisons of the effect of walking durations on the PTI at three insole inner pressure. (**A**) Effect of walking durations on the PTI in T1; (**B**) Effect of walking durations on the PTI in M1; (**C**) Effect of walking durations on the PTI in M2. Data are shown as mean ± standard errors. PTI, pressure time integral; T1, first toe; M1, first metatarsal head; M2, second metatarsal head. *, a significant difference (*P* < 0.05).

The paired t test showed that with an insole inner pressure of 160 mmHg, the PTI obtained with two varied durations was also significantly different (Table 2 and Fig. 2). It revealed that the PTI exhibited with a walking duration of 10 min was considerably lower compared to 20 min in M1 (44.31 ± 4.31 vs. 58.24 ± 5.51 kPa·s, *P* = 0.015). Meanwhile, the PTI obtained at 10 min was also considerably lower compared to 20 min in M2 (47.14 ± 5.27 vs. 65.42 ± 10.30 kPa·s, *P* = 0.047).

**Table 2:** Effects of walking durations on the PTI.

Parameter	Insole inner pressure	Walking duration		Paired t test
		10 min (Mean ± SE)	20 min (Mean ± SE)	*P* value
T1 (kPa s)	80 mmHg	46.40 ± 3.55	56.01 ± 10.57	0.438
	160 mmHg	45.83 ± 5.25	59.25 ± 10.57	0.246
	240 mmHg	40.28 ± 4.72	42.11 ± 8.35	0.852
M1 (kPa s)	80 mmHg	47.55 ± 3.78	50.61 ± 8.51	0.725
	160 mmHg	44.31 ± 4.31	58.24 ± 5.51	0.015*
	240 mmHg	58.17 ± 4.60	58.02 ± 6.02	0.987
M2 (kPa s)	80 mmHg	38.45 ± 3.82	52.96 ± 7.76	0.129
	160 mmHg	47.14 ± 5.27	65.42 ± 10.30	0.047*
	240 mmHg	59.91 ± 4.72	61.79 ± 8.90	0.864

Data are presented as the mean ± standard error. T1, first toe; M1, first metatarsal head; M2, second metatarsal head; *Significant difference (*P* < 0.05).

### Reliability test

The reliability test of the PTI value based on the three insole inner pressures showed that the intraclass coefficient correlation (ICC) values at all locations with a 20 min walking speed were in moderate agreement (0.5 < ICC < 0.75); otherwise, the PTI value at 10 min walking speed were poor moderate (ICC < 0.5), as shown in Table 3.

**Table 3:** Intraclass correlation coefficient on the PTI based on walking three insole inner pressures.

Group	Intraclass correlation coefficient (ICC)
T1 with 10 min	0.202
T1 with 20 min	0.740
M1 with 10 min	0.495
M1 with 20 min	0.575
M2 with 10 min	0.372
M2 with 20 min	0.580

T1, first toe; M1, first metatarsal head; M2, second metatarsal head.

## Discussion

This study aimed to investigate the effects of insole inner pressure and walking duration on PTI. The results demonstrated that with 10 min of walking duration, the insole inner pressure of 80 mmHg significantly reduced PTI compared to 240 mmHg in M1. In addition, the pressure of 160 mmHg also considerably decreased PTI compared to 240 mmHg in M2.

**Figure 3:**
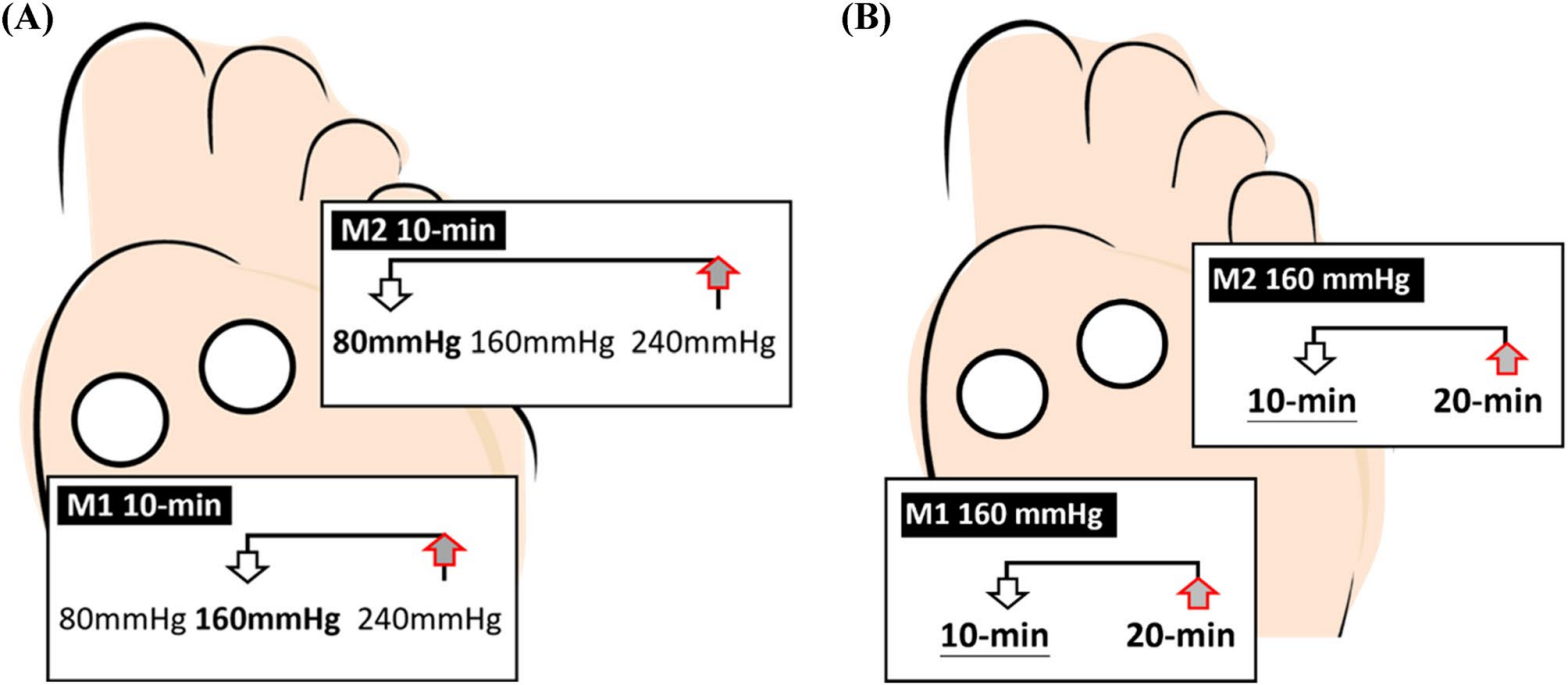
Illustration effect of insole inner pressures and walking durations on the foot regions. (**A**) Effect of the insole inner pressures showed 80 mmHg was lower PTI than 240 mmHg in M2 and 160 mmHg was lower PTI than 240 mmHg in M1 at 10 min. (**B**) effect of the walking durations showed 10 min was lower PTI than 20 min in M1 and M2 with 160 mmHg. M1, first metatarsal head; M2, second metatarsal.

Thus, the study revealed that the effect of the insole inner pressure on the plantar region was significant, indicating that a pressure of 80 mmHg had a more pronounced effect on reducing PTI in M2. In comparison, 160 mmHg demonstrated a more pronounced effect on reducing PTI in M1 (Table 1 and Fig. 2B). The density and thickness of the insole materials prominently influence the plantar pressure^26^. This means that wearing insoles with a higher density results in an elevated PTI owing to the difficulty of effectively redistributing plantar pressure^27^. PTI and PPP are highly correlated across all areas of the foot sole, suggesting that minimizing PTI can substantially increase the ability of the foot to distribute plantar pressure^28^. Specifically, an insole inner pressure of 240 mmHg was not identified as the optimal condition for achieving the highest mesh density after walking (Fig. 3A). This suggests that the combined effect of the adjustment of insole inner pressure and physical activity can significantly contribute to a higher PTI.

Different inner pressures of the insole appear to have different mesh-density properties. An appropriate mesh density in footwear design is pivotal for optimizing the pressure distribution and shock absorption in the foot sole. The inability to disperse and redistribute excessive plantar pressure during walking results in foot pain and increased PTI^29^. Furthermore, an enhanced distribution is important, considering that absorbing and dispersing the forces generated during walking is essential^30^.

Individuals with poor skin properties often experience a reduction in their shock-absorption capacity. This occurs because of the heightened stiffness of the plantar soft tissue, which is characteristic of diabetes^31^. The decline in shock absorption among individuals with compromised skin properties aligns with the findings for high-pressure air insoles. Moreover, deterioration in skin properties, attributed to DFU mostly in the plantar region near the metatarsal heads, contributes to the poor ability of DFU individuals to redistribute plantar pressure on the forefoot^18^. Plantar tissues play a crucial role in absorbing the mechanical loading and redistributing forces during walking. However, increased stiffness in the plantar tissue significantly reduces its ability to tolerate mechanical stresses while bearing weight, thereby increasing the potential for DFUs^32^. These can be considered intrinsic to footwear design aimed at optimizing pressure management and shock absorption while accommodating specific conditions contingent on skin properties. Consequently, it is not recommended for individuals with good skin properties to walk by employing an insole inner pressure set at 240 mmHg.

Additionally, the results also showed that with an insole inner pressure of 160 mmHg, the effects of walking durations of 10 and 20 min on PTI in M1 and M2 also demonstrated considerable differences (Fig. 3B). This indicates that at a medium insole inner pressure with a longer walking duration, the foot's initial shock absorption was less effective, resulting in higher PTI values at the outset. With longer walking duration, the neuromuscular response is affected by sensorimotor adaptation to foot pressure, leading to decreased PTI in M1 and M2^33^. Additionally, longer walking durations allow for more stable and consistent biomechanical patterns, reducing variability, and increasing reliability^34^. Longer walking durations also provide a more comprehensive assessment of the subject's endurance and gait mechanics, leading to a more reliable measurement of walking ability^35^. This finding highlights the nuanced impact that approximately 25% of foot ulcers are located at M1^36^. Similarly, M2, situated between the medial and lateral aspects of the foot, has emerged as a noteworthy site prone to exercise-induced injuries^37^. Meanwhile, air insole material with a pressure of 160 mmHg can also possibly undergo a series of compressions, which can alter its shock-absorbing properties over time.

This investigation highlighted the significant role of insole inner pressure and walking duration on the PTI values. The critical insight from these findings is that walking with an insole inner pressure of 160 mmHg for 10 min can offer a lower risk of foot ulcers. Conversely, walking with an inner insole pressure of 240 mmHg for all durations may increase the risk of foot ulcers. Furthermore, this research contributes to evidence supporting the need for selecting appropriate insole inner pressures to reduce the risk of DFU. Based on the observed variables, it is possible to determine suitable air insole inner pressure for individuals with diabetes, thereby enhancing foot health and preventing the risk of ulcerations.

Nevertheless, this study has several limitations. First, the investigation only involved insole inner pressures ranging from 80 to 240 mmHg, ignoring the potential benefits of lower pressure ranges achievable with softer insoles, which could reduce the PTI during walking. The air in the insole inner pressure density mesh affects the PTI owing to its ability to redistribute plantar pressure. This is because the insole receives different pressure distributions depending on the hardness of the inner insole^26^. A lower hardness insole inner pressure may significantly reduce PPP and PTI^16^. Hence, future studies should extend the tested pressures by examining the effects of pressures lower than 80 and higher than 240 mmHg. This will provide a more detailed understanding of the relationship between insole inner pressure and its impact on PTI, thereby contributing to the development of more effective insole designs for foot health. Second, the PTI measurement only considers PPP in terms of time per frame based on the device frequency**.** A previous study used alternative methods to calculate the PTI, which could consider peak and submaximal pressures^38^. This is a special consideration when calculating PTI because the alternative method contains information on the mechanical loading of the foot sole, which PTI and PPP do not provide. Thus, additional research is needed to compare the different PTI calculation techniques with predetermined factors to assess these results. Finally, this study did not consider the foot posture index (FPI) as a foot type to determine plantar pressure. Variations in foot posture, such as planus or cavus feet, are associated with specific pressure distributions that impact lower limb biomechanics and injury risks^39^. Therefore, additional research is required to compare and understand the differences in FPI in each foot type.

## Methods

**Figure 4:**
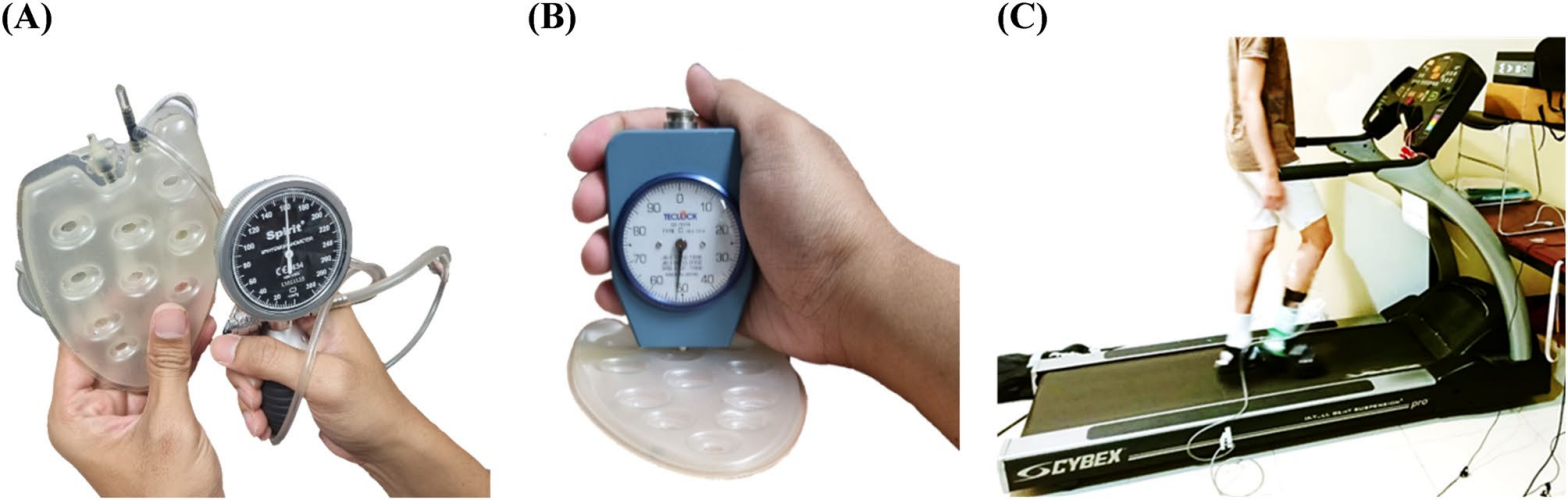
Experimental procedure. (**A**) Air insole measurement; (**B**) Hardness air insole by shore durometer; (**C**) Subjects walked on a treadmill with constant speed equipped with an F-Scan installed in the footwear.

This study employed a repeated-measures design to examine the effects of two walking durations (10 and 20 min) and three air insole inner pressures (80, 160, and 240 mmHg) on PTI, as shown in Fig. 4A. The hardness values of the air insoles at different pressures were determined using a GS-701N Shore durometer (Teclock Co., Ltd., Nagano, Japan)^40^. The yields exhibited shore values of 51.7 ± 1.5, 54.7 ± 0.6, and 57.7 ± 0.6 at 80, 160, and 240 mmHg, respectively, as shown in Fig. 4B. In addition, this study selected three inner insole pressures to ensure appropriate stiffness for walking^9^. Based on the American Guidelines and the American Diabetes Association, a walking speed of 3.6 mph was chosen, as it is commonly used^41,42^. This study was part of a larger project investigating the effects of different insole inner pressures and walking durations on plantar soft tissue properties^9^.

### Participants

The study involved 13 healthy participants (seven men and six women) aged 21–39 years. Participant characteristics (mean ± SD) included age of 27.0 ± 7.3 years, body weight of 56.0 ± 7.9 kg, body height of 165.8 ± 8.4 cm, and Body Mass Index of 20.3 ± 1.7 kg/m^2^. The required criteria were shoe size for men (41–43) and women (36–38) with body weights less than 80 kg and right-leg dominance. The Central Regional Research Ethics Committee approved this study at the China Medical University, Taichung, Taiwan (CRREC-111-017). All participants received information about the study's purpose and procedures, as a prerequisite for their involvement in the research. All the participants provided written informed consent. The participants were assured that their data would be kept confidential. The responses were anonymized, and a confidentiality agreement was established, allowing respondents to withdraw from the study at any point, based on the Declaration of Helsinki.

### Experimental procedure

The participants were instructed to remove their socks and shoes and lie down for 20 min to minimize the impact of prior weight-bearing activities on muscle fatigue and plantar pressure. The specific walking procedure was as follows:

- An insole inner pressure of 80 mmHg and a walking duration of 10 min
- An insole inner pressure of 80 mmHg and a walking duration of 20 min
- An insole inner pressure of 160 mmHg and a walking duration of 10 min
- An insole inner pressure of 160 mmHg and a walking duration of 20 min
- An insole inner pressure of 240 mmHg and a walking duration of 10 min
- An insole inner pressure of 240 mmHg and a walking duration of 20 min

The participants randomly selected the insole inner pressure and walking duration each week. They were asked to wear commercial shoes (Hsin He Hsin Co., Ltd., Taichung, Taiwan) and walk on a treadmill (Cybex DE-20427 A, Cybex, Taoyuan, Taiwan), as shown in Fig. 4C. The inner pressure insole was made of a thermoplastic polyurethane material. It is located inside shoes to cover the forefoot area to specifically target and alleviate the high plantar pressures and shear forces concentrated in this region. This is crucial because high plantar pressure and shear force are critical factors for the development of metatarsalgia and other forefoot-related conditions. By focusing on the forefoot, air insoles can provide localized pressure relief and improve shock absorption^8^. It further effectively reduces the strain on this vulnerable area while also enhancing overall gait stability and comfort.

PTI was determined using an F-Scan plantar pressure measurement system (Tekscan Inc., South Boston, MA, USA). This system has a sensor with 960 sensing elements, where the dimensions of each element are 5.08 × 5.08 mm. Before walking on a treadmill, the participants walked for 3–5 min to familiarize themselves with the shoes^43^. The sensor was calibrated according to the manufacturer's guidelines to minimize measurement errors^43^. Herbert-Copley et al.^44^ performed an extensive analysis of the F-scan system to evaluate its performance over extended periods. Despite variations in the total force values over time, the F-Scan system consistently provided accurate evaluations of the pressure profiles and center-of-pressure trajectories. This indicates that the sensor's ability to measure the relative pressure distribution remains reliable, even over extended periods^44^.

### Data analysis

**Figure 5:**
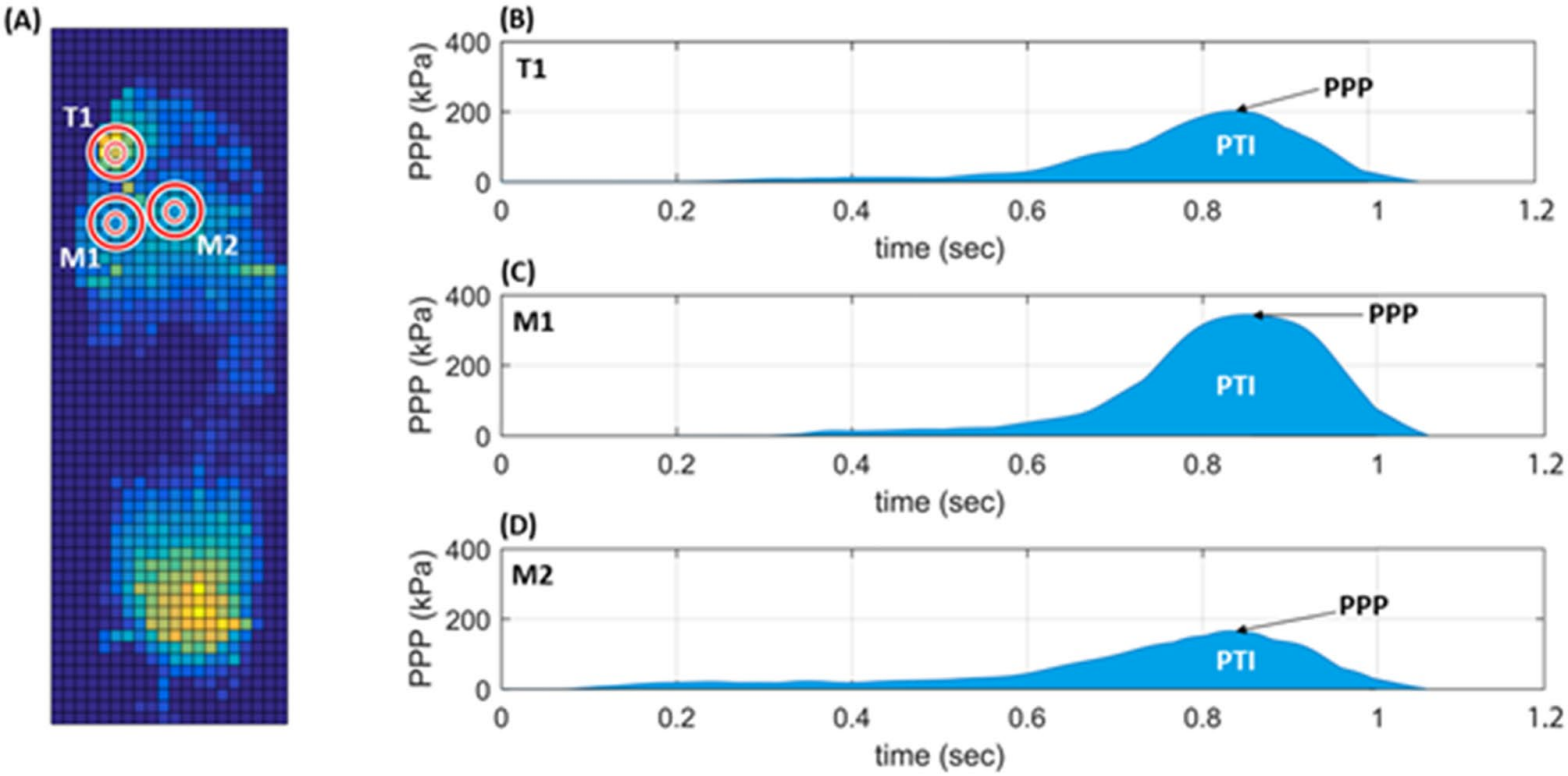
Illustration of Plantar Pressure and PTI Data. (**A**) Plantar pressure data; (**B**) PTI data in T1; (**C**) PTI data in M1; (**D**) PTI data in M2. PTI, pressure time integral; T1, first toe; M1, first metatarsal head; M2, second metatarsal head.

PTI values were obtained from three intermediate steps from the last minute of each trial. All data were analyzed using MATLAB 2022b (The MathWorks, Natick, MA, USA). Data were filtered using a 2nd Butterworth low pass filter applied backward and forward, with a cut-off frequency of 150 Hz. This study focused on selecting three regions of the forefoot with a heightened risk of developing DFU: the first toe (T1), first metatarsal head (M1), and second metatarsal head (M2). The PTI data were determined from the plantar pressure in the contact area of a particular foot sole (5 × 5) F-Scan sensor pixels (645.2 mm^2^)^45^. This approach calculates the PTI by summing the peak pressures per sample (Fig. 5). The PTI for the plantar area was determined by adding the products of the peak pressure and duration of the sample, using Eq. 1^38^:

1 $$PTI=\sum_{i=1}^{N}PPi \times \Delta t$$

where PPi is the peak pressure at the i-th time, N is the total number of frames, and $\Delta t$ is the time sample duration.

### Statistical analysis

The PTI values are presented as the mean ± standard error. A multivariate analysis of variance (MANOVA) test was used to analyze the main effects of the insole inner pressure and walking duration, as well as the interaction effect between the insole inner pressure and walking duration. One-way analysis of variance (ANOVA) with a least significant difference (LSD) post-hoc test was used for pairwise comparisons of the PTI between the three insole inner pressures and two walking durations^9,46^. The differences in PTI between the insole inner pressure and walking duration were examined using a paired t test. All statistical analyses were performed using SPSS version 22 (IBM, NY, USA) with a significance level of 0.05. Additionally, the intraclass correlation coefficient (ICC) was calculated, assuming separate data classes, and the three different insole inner pressures were instructed to perform the relative tests.

### Ethical experimentation

Studies involving humans were approved by the Central Regional Research Ethics Committee of China Medical University, Taichung, Taiwan (CRREC-111-017). The studies were conducted in accordance with local legislation and institutional requirements. All participants provided written informed consent to participate in the study.

## Data Availability

Research data supporting this publication are available from the corresponding author upon request.
